# Effectiveness of Swirl Water Mist Nozzles for Fire Suppression

**DOI:** 10.3390/ijerph192316328

**Published:** 2022-12-06

**Authors:** Natalia Kraus-Namroży, Dorota Brzezińska

**Affiliations:** Faculty of Process and Environmental Engineering, Lodz University of Technology, Wolczanska 213 Street, 90-924 Lodz, Poland

**Keywords:** people safety, evacuation, low pressure water mist nozzle, fire extinguishing, sprinkler, water mist

## Abstract

Water mist nozzles are becoming increasingly popular as water extinguishing devices in buildings, increasing people’s safety during evacuation. There are questions as to whether or not they are as effective as conventional sprinkler systems. The measurement of the size distribution of the created droplets is one of the components for determining the extinguishing effectiveness of water mist nozzles. The project’s goal was to look into the characteristics of the atomized stream produced by a swirl water mist nozzle. The general properties of the tested nozzle are covered in the article. The measurement technique and test stand were given. The collected data was compared to data from the literature. The results are displayed as graphs that depict the distribution of mean droplets with a quantitative volume that varies with pressure and proximity to the test nozzle. The results proved the extinguishing and cooling capabilities of hot fire gases in the water stream created by the tested nozzle.

## 1. Introduction

The fixed water extinguishing system market/industry is constantly developing. Systems may differ in the size of droplets generated and in their operating pressure, just like sprinkler systems and low-pressure and high-pressure water mist systems do. There is no one-size-fits-all system that will extinguish every fire and is applicable to all operating conditions [1]. To select an adequate extinguishing system, the hazard must be properly classified. Classifying a fire hazard on a building as LH low hazard, OH ordinary hazard or HH high hazard depends on the fire load and on the particularities of the designated space [2]. Sprinkler systems are often used to ensure the protection of life and property in buildings [1]. These are designed to detect and extinguish a fire in its initial stages of development or to limit its spread until the arrival of firefighting units. There are several standards according to which sprinkler systems should be designed: the American NFPA standards [3]; the German VdS guidelines [4]; or the Polish PN-EN 12845 [2], for example. The rapidly developing water mist systems are a competitive alternative to sprinkler systems. Their construction is similar to that of traditional sprinkler systems, with the main difference being its specially designed water mist nozzles. Automatic fog nozzles are mounted on pipelines connected to sectional valves. Water is fed into the system by a pump from a water tank; water pressure will be determined by the type of water mist nozzle in use—high or low pressure. Due to the special nozzles, water mist systems require much smaller amounts of water than sprinkler systems [5]. As a result, water mist systems have a significantly smaller pipe diameter (which, consequentially, takes up less space in the ceiling and reduces the strain on the ceiling structure) as well as smaller extinguishing water tanks. This is also reflected in the attenuated costs of firefighting installations in the building. The difference also lies in the mechanism of fire extinguishing. 

The fire extinguishing process is greatly affected by the reaction of the water particles with the fire. The minuscule droplets, produced by the water mist nozzles, effectively limit a fire hazard’s growth by absorbing heat, wetting and cooling the fuel surface [6,7,8,9]. Water mist creates an extensive cooling surface, and as soon as water droplets penetrate the fire, they transform into water vapor, thus cooling the flame. When the lower limit of the adiabatic temperature of the flame is reached, the combustion reaction of the fuel–air mixture ends, and the fire is extinguished [10]. This is also achieved by reducing the surface temperature of the burning material in which case the heat absorption rate of the atomized water particles must be greater than the heat produced from the flammable reaction. The fuel cooling is also achieved by lowering the amount of fuel–air mixture above the surface of the burning material below the flammable limit. The heat transferred from the flame to the fuel occurs through convection currents and thermal radiation, while the heat taken from the burning fuel by the water particles will transform water into steam [10]. 

Flame suppression also occurs through a local decrease in oxygen concentration levels due to displacement by water vapor [6,7,8,9]. This phenomenon affects both the combustion zone and the surrounding environment, areas where water vapor is formed most rapidly due to high temperatures [11]. This mechanism plays its most significant role during firefighting when in an enclosed space. In the case of an open space, a factor that affects the speed and effectiveness of fire suppression is the air exchange caused by wind or ventilation—because of it, the process is slowed down [11]. The oxygen displacement effect also explains why it is easier to suppress a “larger” fire than a “smaller” one. The terms “larger” and “smaller” refer to the effect of the fire on the average temperature and oxygen concentration in the room during water mist release. The former, unlike the latter, gives more heat to the room in the initial stages, so more heat is available for the process of evaporation of small water droplets. Large fires will also reduce the oxygen concentration in the fire environment to a level where the combustion efficiency will be reduced prior to the introduction of water mist [12]. The water mist creates a barrier to prevent further thermal decomposition of the fuel surface, including unscathed surfaces. Suppressing thermal radiation ensures that the flame does not consume the burning material with the same intensity. It also blocks the expansion of the area occupied by the fire and reduces the growth of fuel vapor and the intensity of the pyrolysis phenomenon [11]. Radiation blocking is correlated by and large with the diameter of the water droplet and the droplet’s mass density. A certain volume of water will provide a more effective barrier against heat radiation if dispersed in very small droplets in a dense stream rather than in a scattered stream with larger droplets [13,14]. Due to the large surface area of the droplets produced, water mist can absorb large amounts of heat and therefore provide effective cooling of the combustion zone [15].

Water extinguishes a fire by three main mechanisms, namely, cooling the combustion surface, cooling the burning area and volumetric displacement of combustible gases and oxygen. By lowering the temperature of the combustion surface, the rate of chemical reactions is reduced, contributing to a lower rate of both heat release and heat transfer from the flames to the surface. This also slows or even stops the production of volatiles [16]. These are the main mechanisms for extinguishing solid fuels, or Group A fires. Some of the heat generated from the combustion reaction is absorbed by the water particles that thereafter evaporate, and in turn, the flame temperature lowers until extinguished. Through the process of converting liquid water into gas, a large amount of steam is produced, further contributing to the suppression of the flame [17,18]. It is understood that the droplet size produced by the extinguishing system is the main factor in the fire extinguishing process [9].

Scientific research has shown that the degree of atomization of water has a significant impact on the effectiveness of firefighting operations [19]. The safety of the occupants of a facility should be the main argument for the use of water fixed extinguishing devices, [5] as they are the only systems that have a completely inert effect on the human body during a fire [20].

A water mist is a water spray generated by a water mist nozzle for which the D_v0.90_ total volumetric atomization of water droplets is less than 1000 microns when operated at minimum pressure [21]. The increasing dependence on water mist nozzles increases the importance of achieving the best-suited droplet size, leading to the most efficient extinguishing effect [20]. However, it is difficult to unequivocally state and systematize the extinguishing effectiveness of any droplet size, since it depends on the type of fire (group A, B, F). 

Droplets larger than 1 mm are generated by sprinklers [9]. This size makes it easier for the droplet to reach the ignition source and, therefore, increases the level of heat absorption, cooling down the flame area. On the contrary, the momentum and mass of droplets generated by water mist systems may not be sufficient to penetrate deep into the flame area. Although evaporation is the main factor preventing the water mist droplets from reaching the flame, by using higher inlet pressure at the nozzle, the water mist droplets are still able to penetrate the flame area [22].

Droplet size distribution refers to the range of the droplet size contained in representative samples measured at specific locations. The NFPA 750 standard [23] divides the droplets produced by the water mist nozzles into three classes. Class one water mist is characterized by droplets at 90% of the volume (D_v0.9_) of 200 μm or smaller; class two water mist has a D_v0.9_ of 400 μm or less; and class three water mist has a D_v0.9_ value greater than 400 microns. One may ascertain that droplet size for fire suppression is strongly dependent on many factors, such as the properties of combustible materials, the complexity of the room and the size of the fire. The droplet size distribution that is most effective in extinguishing one fire scenario will not necessarily be the best for other scenarios. There is no single water mist droplet size distribution that fits all fire scenarios. 

To determine the appropriate droplet size for fire suppression, a wide range of studies have been conducted under different fire conditions [24,25]. There are several theories on the optimal droplet size for firefighting effectiveness [19]. The larger the spray, the smaller the droplets, which better cools the combustion zone. On the other hand, if the water droplet diameter is too small, there is a high probability of it not reaching the source of the fire. Droplets that are too small evaporate quickly and only reduce the combustion zone [20]. Schremmer’s research shows that atomized water mist particles of 10 to 400 μm will achieve an extinguishing effect as much as ten times greater than conventional nozzles generating droplets larger than 1000 μm (sprinklers) will [26]. The method described by Schremmer [14] is characterized by a very rapid decrease in the cooling down of the burning material, significantly reducing the access of oxygen to the fire source and the (so-called) radiant heat transfer. This will reduce the number of combustion products, including smoke release. In another postulation, an average droplet with a diameter of 300 μm was adopted as the optimal dimension to cool the gas phase using dispersed streams [8]. The Swedish and Finnish Fire Research Council, in turn, expressed the opinion that nozzle-generating droplets with an average diameter of 400 μm should be used to achieve the highest cooling effect [8]. According to the research of the Swedish Fire Research Board, Paul Grimwood states that water mist with an average droplet diameter of 200–400 μm is the best fit for firefighting purposes [7,15]. In contrast, for the suppression of fire and cooling fire gases inside wooden structures, a droplet range of 100–300 μm seems to be the most suitable [20].

The liquid jet breakup mechanism and droplet formation are thoroughly discussed in existing investigations [19,27,28]. The characteristics of the spray jet are divided according to their microstructural and macrostructural properties. These are strictly dependent on the technical specifications of the nozzle, its distance and mounting height [15]. Macrostructural characteristics include the angle of spray and the range of the spray jet, which describes its external shape. Microstructural characteristics include the average droplet diameter, its distribution in the horizontal plane (also known as the spray spectrum), maximum and minimum diameters, and the specific surface area of the droplets. A detailed discussion of the above parameters can be found in [19,27,28,29].

Unlike the sprinkler system, which only affects the heat of combustion, the water mist system acts upon two of the three elements of the triangle of combustion—specifically, the oxidant and the heat of combustion [11]. Water mist can be used to extinguish solid fires and flammable liquid fires. 

Until the end of 2020, water mist installations in Poland were designed based on European standards, e.g., Great Britain (BS), Germany (VdS) or the American standards (NFPA). Since December 2020, the Polish standard PN-EN14972 [21] has been in effect. Unlike the standards for sprinkler systems that give specific design guidelines, the standards for water mist systems describe, in general terms, the principles for designing these systems. According to its guidelines, a water mist installation is a specific, unique solution that must be tested for every single installation [21]. Consequently, any new water mist nozzle used in an extinguishing system must be certified. It is necessary to carry out tests to determine the water mist’s spray spectrum, reach and maximum height for a given water mist nozzle installation.

In this article, the parameters of the microstructure of the spray jet match those produced by a swirl-type water mist nozzle, in particular, the average volumetric diameter of the droplets Dv are equivalent. Based on the Dv, an attempt was also made to compare the new nozzle with other existing water mist nozzles and sprinklers previously studied by a team of researchers led by Hengrui Liu [9]. This team conducted a numerical analysis of the extinguishing mechanisms of the sprinkler and water mist systems. For each system, three nozzles that generate different droplets were selected to study their fire extinguishing efficiency. For the water mist system, nozzles generating droplets of 100, 150 and 200 μm were examined. The sprinklers tested produced droplet sizes of 1000, 1750 and 2000 μm. A comprehensive analysis of temperature, velocity, relative humidity level and oxygen concentration level was done for each case. The team found that, for mist installations, the main extinguishing mechanisms are latent cooling, volumetric displacement and the dilution of oxygen and fuel by evaporation—which is more easily achieved with smaller droplets. Regarding droplets generated by sprinklers, a direct heat removal from the flame area plays the most important role in fire suppression, while the effect of evaporation is an insignificant contribution to overall fire suppression. Studies have established that, under the same conditions, water mist systems perform better in terms of suppression time. They also suggest that water mist produces higher relative humidity levels than sprinklers, making the fire environment less suitable for sustaining fire and diminishing the risk of reignition. By comparing three different droplet sizes, they found that the 100 μm droplet is the most effective size, reducing suppression time by 4.26% when compared to the 300 μm size, while the 200 μm droplet only reduces suppression time by 11.9%. According to the investigation, the difference between the selected droplet size in the water mist system is not significant, so aiming for extra small droplets may not be necessary. On the other hand, in sprinkler systems, the water droplet size has a big impact on its extinguishing capacity, and for this reason, it is important to use the right sprinkler to improve the overall performance of the system. 

The goal of this article was to determine the extinguishing efficiency of the swirl water mist nozzle in consonance with PN-EN 14972. The presented method was based on the size of the water mist droplets. This assumes a comparison of the nozzle’s droplet size with the results achieved by the Liu research team presented above. This is an economically valuable method and could give a first answer as to whether the designed nozzle would be effective. If the analysis shows correct extinguishing qualities of the tested nozzle, in the next step, real scale tests according to the standard should be performed.

## 2. Materials and Methods

In this section, the swirl water mist nozzle is described. An AWK D analyzer was used to measure the spray spectrum of the tested nozzle. The tested nozzle was placed 2.3 m above the analyzer. The average diameters were estimated based on the obtained measurements, allowing the extinguishing efficiency to be evaluated.

### 2.1. Research Subject

**Figure 1:**
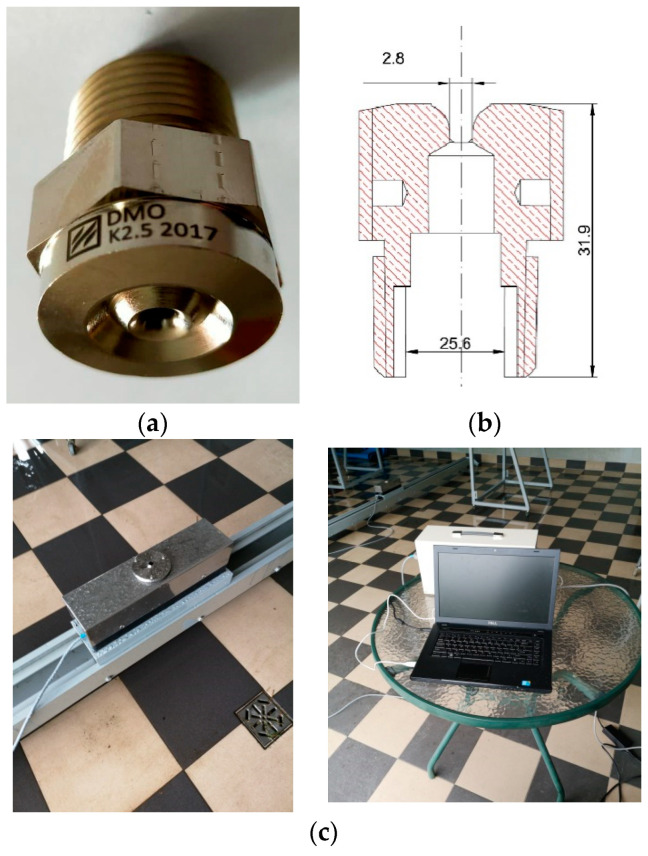
Low-pressure swirl water mist nozzle (**a**) photo, (**b**) technical drawing [mm], (**c**) AWKD Analyzer.

The object of research was the low-pressure swirl atomizer shown in Figure 1a. This water mist nozzle is characterized by an X-shaped swirler mounted in a 22-mm long swirl chamber. The outflow coefficient K for the tested nozzle is 2.5. The technical drawing is shown in Figure 1b.

The aim of the study was to measure water mist nozzle spray spectrum. The obtained results determined the spray spectrum characteristics of the tested water mist nozzle, as well as its extinguishing efficiency. The AWK D water mist droplet size and shape analyzer presented in Figure 1c was used to carry out the test. The spray spectrum of the water mist nozzle was measured at a minimum of 0.4 MPa, medium of 0.6 MPa and maximum of 0.8 MPa working pressure.

### 2.2. Description of the Measurement Method

**Figure 2:**
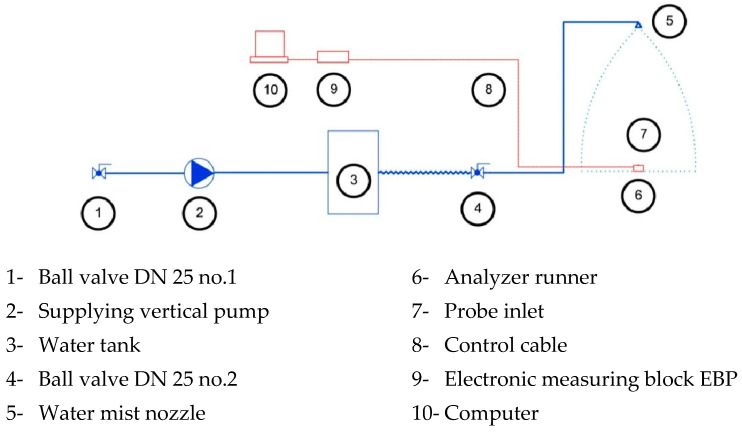
Diagram of the test.

The AWK D analyzer consists of a probe with a photoelectric converter connected to the electronic measuring block EBP with a 20-m cable. The measuring range of the analyzer is 50 mm–4 mm. The electrical impulses formed by the EBP are proportional to the diameter of the droplets. As shown in Figure 2, the EBP (9) is connected by a USB 2.0 cable (8) to a computer (10). The measured results were saved in the computer’s memory in electrical units and converted into physical units, according to the parameters specified by the manufacturer. The results were presented on screen, and the device was controlled using the keyboard. The probe (7) has three variable drop inlets that are used depending on the concentration and size of the droplets. The droplet concentration depends on the liquid flow rate in the atomizer and the distance from the atomizer (5) to the probe (7). The infrared radiation beam from the sensor is scattered by droplets passing through the measurement zone. Each droplet has an electrical impulse proportional to its size. The set of droplets was originally measured in 4096-dimensional classes. After the measurement, the set of drops is converted into 256-dimensional classes available to the user. The adopted study method contains an error due to the AWK system’s maximum total measurement error of 2.5%.

Based on the measurements, the characteristics that clearly defined the set of particles were calculated. The presented method of data processing made it possible to quickly obtain results in a systematized form using numerical methods. Based on the recorded results, the average diameters occurring in each zone of the area of the particle collection as well as in the entire set were calculated.

### 2.3. Description of the Tested Area

The test was carried out in a laboratory room with the following dimensions: 5 m (length) × 7 m (width) × 2.85 m (height). A scheme of the tested area is shown in Figure 2. 

**Figure 3:**
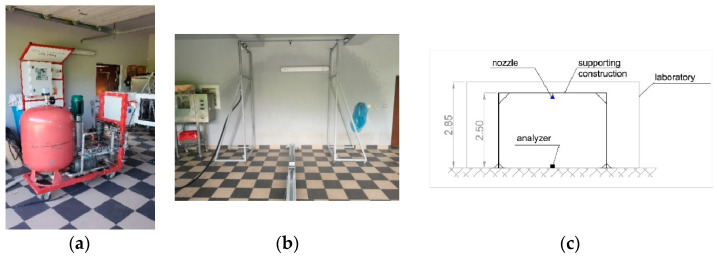
(**a**) View of the pump set; (**b**) View of the tested area—mobile setting; (**c**) Front view of the laboratory.

Water was supplied to the system using a vertical pump (2) with a capacity of 116 L/min and a maximum pressure of 1.58 MPa, as shown in Figure 2 and Figure 3a. A DN52 fire hose was used to supply the pump. During the measurements, the pump (2) supplied water through a 25-mm diameter rubber hose to a DN25 ball valve no.1 attached to the mobile structure shown in Figure 3b and pumped through the water tank (3) supply through ball valve no.2 water mist nozzle (5). As shown in Figure 3c, the sprayer was mounted 2.5 m above the floor. The measuring probe was 0.2 m above the floor level and measured the droplets from a nozzle mounted 2.3 m above the floor.

### 2.4. Test Procedure

**Figure 4:**
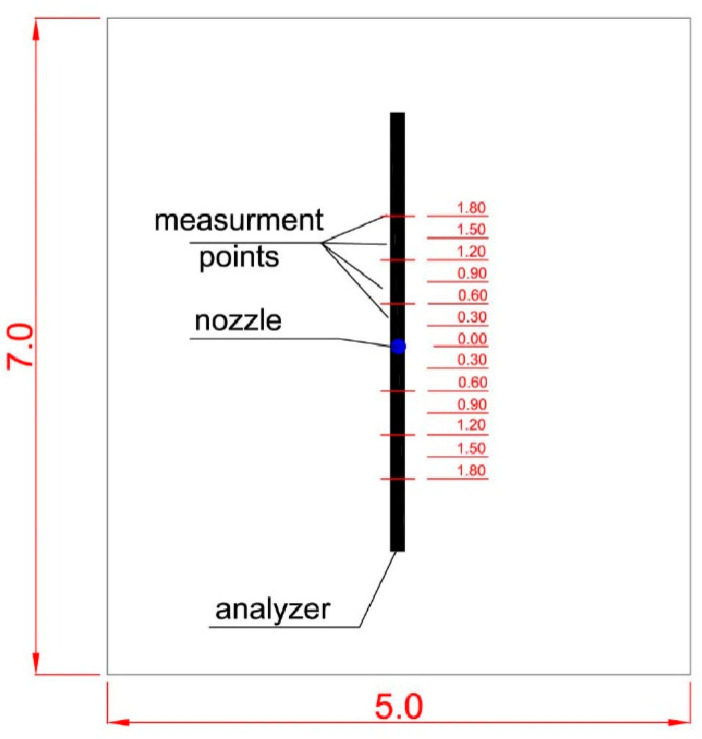
Top view of the tested area with measuring points.

Spray spectrum measurements were carried out at 3 pressures: 0.4 MPa, 0.6 MPa and 0.8 MPa at 18 °C and 55% humidity. The measurement was carried out in accordance with CEN/TS 14972. As shown in Figure 4, 7 measurements were made along the y-axis of the spraying field by moving the probe every 0.3 m over a length of 1.8 m. The measuring probe started mensuration as soon as the START button was pressed, stopping automatically after 30 s.

## 3. Results and Discussion

The mean droplet volume diameters Dv, determined by the AWK D program, were used to describe the results and to draw an analysis. These data are considered the most reliable for the analysis of firefighting effectiveness [15].

The results were obtained from the AWK D analyzer measurements, thereafter calculating the average volume diameters of the droplets Dv for each measurement. The method of calculating the Dv (by the analyzer) is expressed by the relation (1):

(1) $$dv=\sqrt[{3}]{\underset{i}{\sum }A_{i}*{\left(d_{i}\right)}^{3}}\;A_{i}=\frac{\sum_{i}n_{ij}}{\sum_{j}\sum_{i}n_{ij}}$$

$A_{i}$—number of particles in the *i*-th measurement class for all zones of the study area.

*d*—diameter of the droplets;
*n*—number of measured droplets;
*i*—current number of dimension class;
*j*—current measurement number.

The obtained droplet sizes at the measurement points at each pressure value are shown in Table 1. 

**Figure 5:**
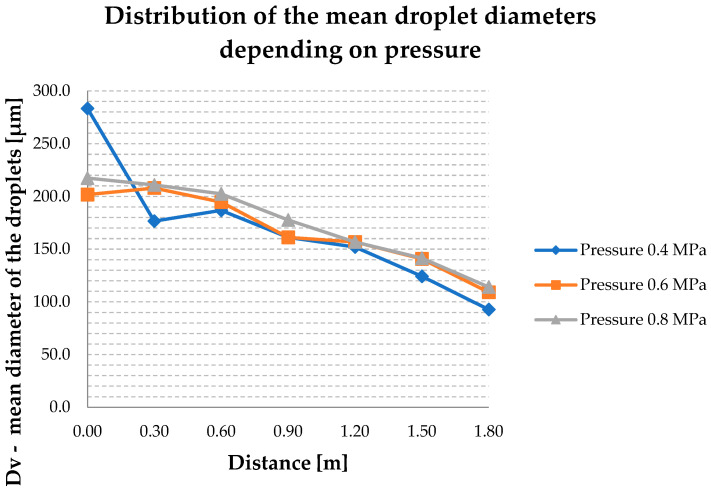
Radial distribution of mean droplet diameters at 0.4 MPa, 0.6 MPa and 0.8 MPa.

The radial distributions of the mean volume diameters of the droplets Dv along the y axis are shown in Figure 5 for a radius of 0.0 m, 0.3 m, 0.6 m, 0.9 m, 1.2 m, 1.5 m and 1.8 m.

The radial distribution of Dv is very similar at all measuring points at any pressure as is shown in Figure 5. The differences mostly do not exceed 20 μm. It can be assumed that, with increasing pressure, the mean diameter Dv increases at each of the measuring points. As the graph shows, the highest average diameters are at a pressure of 0.8 MPa. However, in all tested distributions, Dv has a maximum of 283.4 μm at the point of 0.0 m at a pressure of 0.4 MPa. Then it rapidly decreases to a value of 176.6 µm at a distance of 0.3 m from the nozzle. As the distance from the nozzle increases, the decrease rate of the Dv is much smaller and is the lowest at a pressure of 0.4 MPa at 1.80 m, corresponding to 92.7 μm. At a distance of 1.2 m from the nozzle, the value of Dv at a pressure of 0.6 MPa and 0.8 MPa is the same, 156.7 μm. Moreover, at a distance of 0.9 m from the nozzle, the mean droplet diameter is 161.2 μm and is the same for 0.4 MPa and 0.6 MPa. For a pressure of 0.6 MPa, the distribution ranges from 201.7 μm to 109.1 μm, reaching its minimum at the point of 1.8 m. The radial distribution of the droplet size obtained at the pressure of 0.8 MPa is relatively uniform—the maximum size of 217.4 μm is recorded at 0.0 m, and the minimum distance is 1.8 m from the nozzle.

In order to carry out a more detailed analysis of the results obtained, the values of the total mean droplet diameter $\bar{Dv}$, which is the ratio of the sum of the mean volumetric diameters at each measurement point of the 21 measurement points, and the standard deviation *σ_Dv_* of the mean volumetric diameter of the droplets were determined.

The total mean volume diameter of the droplets $\bar{Dv}$, in μm, is expressed by the formula:

(2) $$\bar{Dv}=\frac{\sum_{i=1}^{n}D_{vi}}{n}$$

where:

*n*—number of measurements points equal 21.

The result based on the obtained data $\bar{Dv}$ = is 170 μm.

In the next step, the standard deviation of the mean volume diameter of the droplets *σ_Dv_* was calculated (3):

(3) $$\sigma_{Dv}=\sqrt{\frac{\sum_{i=1}^{n}{\left(D_{vi}-{\bar{D}}_{v}\;\right)}^{2}}{n}}$$

The standard deviation for the entire area under consideration is approximately 41 μm.

The indicator of the proximity rate to the optimal diameter of the *WSO*, in μm, was also determined (4):

(4) $$WSO=\sqrt{\frac{\sum_{i=1}^{n}(D_{vi}-D_{vopt)}{}^{2}}{n}}$$

where:

*WSO*—the index of the proximity rate to the optimum diameter;
*D_vopt_*—assumed based on the available literature [9], the optimal mean droplet volume diameter for fire extinguishing efficiency is 100 μm.

**Figure 6:**
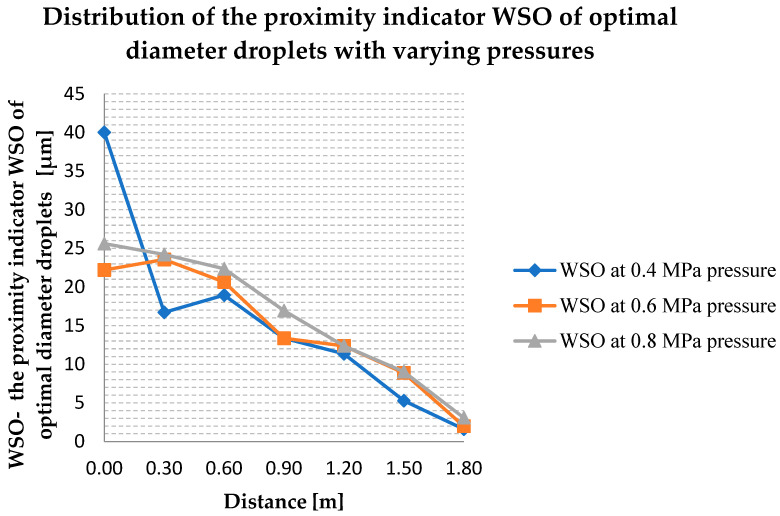
Distribution of the proximity indicator *WSO* of optimal diameter droplets with varying pressures.

The *WSO* index shown in Equation (4) indicates to what extent the measured diameters are close to the value assumed to be optimal in terms of extinguishing efficiency. The value of WSO for the entire study area is approximately 82 μm. Performing an analysis of the distribution of values for this indicator, it can be seen that the diameters of the droplets closest to the optimum were obtained at the measurement point of 1.8 m—the average *WSO* = 2 μm, and the least close axially under the water mist nozzle at the point 0.0, where the average *WSO* = 29 μm. Analyzing Figure 6, it can be concluded that the WSO decreases with the distance from the nozzle, so the droplets generated by the water mist nozzle on the outside of the circle are close to optimal in terms of extinguishing efficiency. 

The total volumetric diameter of the droplets $\bar{Dv}$ obtained in the measurements was 170 μm, which was of the same order of magnitude as that considered optimal from the point of view of extinguishing efficiency (as 100 μm) previously indicated by Liu [9].

In summary, it should be stressed that the presented studies, although they have allowed for analysis of the effectiveness of swirl water mist nozzles for fire suppression, have not covered the whole subject. In future research, the authors hope to conduct a full-scale fire test according to new standard PN-EN 14972 in order to accurately determine the effectiveness of the tested low pressure water mist nozzle. Tests based on this standard would make it possible to compare the results obtained, to evaluate the analysis carried out and to determine the extinguishing efficiency of the average droplet diameters obtained in the sprayed liquid. It would also be advisable to carry out an analysis of the influence of other factors, such as flow rate, on the quality of the spray obtained, with particular attention to the size of its droplets.

## 4. Conclusions

Based on the conducted analysis of the swirl water mist nozzle, the following conclusions were formulated:

- The tested water mist nozzle generates drops smaller than 1 mm in 90% of its volume, so it is suitable for use in water mist extinguishing systems. It can be used to extinguish and control the fire.
- The studies have shown that the radial distribution of the mean diameter of the droplets is uniform. The Dv values are very similar at the same measuring points at different pressures. This demonstrates the uniformity of sprinkling when extinguishing a fire. From the standpoint of the manufacturer or designer, this is critical information that influences how the nozzle is applied.
- The mean droplet diameter (Dv) decreases with increasing distance from the nozzle axis. The total volumetric diameter $\bar{Dv}$ is close to the optimum volume mean diameter of the droplet Dv opt equal to 100 μm, assumed based on the literature survey, and is 170 μm.
- It has been demonstrated that the *WSO* index of the proximity rate to the optimum diameter has the lowest value for r = 1.8 m, so at this distance from the axis of the water mist nozzle, the droplet sizes are closest to the optimal values in terms of extinguishing efficiency. In order to ensure extinguishing efficiency, the nozzle spacing should be considered when designing and installing tested water mist nozzles.

The presented method of the swirl water mist nozzle allows for a reasonably quick and economically sensible review of water mist nozzles, suggested by the article’s authors, for use in all situations where the water mist system is planned for installation or a new model of nozzles needs to be verified in accordance with PN-EN 14972 standard.

## Figures and Tables

**Table 1:** Mean volume diameter of the nozzle droplets at 0.4 MPa, 0.6 MPa and 0.8 MPa.

Distance	Dv—Mean Diameter of the Droplets [μm]	Dv—Mean Diameter of the Droplets [μm]	Dv—Mean Diameter of the Droplets [μm]
	Pressure 0.4 MPa	Pressure 0.6 MPa	Pressure 0.8 MPa
0.00	283.4	201.7	217.4
0.30	176.6	208.0	210.9
0.60	186.8	194.6	202.5
0.90	161.2	161.2	177.6
1.20	152.0	156.7	156.7
1.50	124.2	140.7	141.4
1.80	92.7	109.1	114.1

## Data Availability

The data that support the findings of this study are available from the corresponding author upon reasonable request.
